# Synthesis and Characterization of the Metal–Organic Framework CIM-80 for Organic Compounds Adsorption

**DOI:** 10.3390/ma15155326

**Published:** 2022-08-02

**Authors:** Leidy Figueroa-Quintero, Enrique Vicente Ramos-Fernandez, Javier Narciso

**Affiliations:** 1 Laboratorio de Materiales Avanzados, Departamento de Química Inorgánica, Instituto Universitario de Materiales de Alicante, Universidad de Alicante, Apartado 99, 03080 Alicante, Spain; leidy.figueroa11@ua.es (L.F.-Q.); enrique.ramos@ua.es (E.V.R.-F.); 2 Instituto de Investigación Sanitaria y Biomédica de Alicante (ISABIAL), 03690 Alicante, Spain

**Keywords:** metal–organic framework, COV, adsorption, CIM-80

## Abstract

Metal–organic frameworks (MOF) are a new type of porous materials that have great potential for adsorption of voltaic organic compounds (VOCs). These types of materials composed of metal ions and organic ligands are easy to synthesize, have high surface areas, their surface chemistry can be adjusted to the desired application, and they can also have good chemical and thermal stability. Therefore, this work focuses on the synthesis of a highly hydrophobic MOF material called CIM-80, a porous material that is made up of the Al^3+^ cation and the mesaconate linker. This MOF has a B.E.T. of approximately 800 m^2^/g and has potential applications for the adsorption of hydrophobic organic compounds. However, its synthesis is expensive and very dirty. Therefore, we have studied the synthesis conditions necessary to achieve high synthesis yields (85%) and materials with high crystallinity and accessible porosity. To achieve these results, we have used urea as a mild deprotonation reagent and modulator as an alternative to NaOH, which is traditionally used for the synthesis of this MOF. Once the synthesis of this material was controlled, its adsorption/desorption behavior of water and organic compounds such as toluene, cyclohexane and m-xylene was studied by means of vapor adsorption isotherms. The results show the hydrophobic character of the material and the greater affinity the material has toward aliphatic compounds than toward aromatic ones, with toluene being the most adsorbed compound, followed by cyclohexane and m-xylene.

## 1. Introduction

Volatile organic compounds (VOCs) are a type of pollutant that can have a negative impact on the environment and humans [1,2]. Emissions of these compounds are mainly generated by the transport and industry sectors; however, they can also occur in the tertiary sector. This includes residential, commercial and service activities. This has been of great concern worldwide, and efforts have been made to eliminate the emission of these compounds, which, in turn, has led to years of research into methods to reduce these emissions. Among the alternatives studied, adsorption is one of the most promising techniques for the capture of VOCs, since it has interesting characteristics in environmental terms, thanks to its cost-effectiveness, flexible operation and low energy consumption. Thus, the adsorption of this type of compound on porous materials has been studied as a promising alternative [3,4,5], which implies a very important challenge: the development of adsorbents selective toward specific adsorbates and thus overcoming the limitations of conventional adsorbents such as zeolites [6,7,8], activated carbons [9,10,11,12,13] and silica [14,15]. 

Regarding activated carbons, Lillo et al. [16] in one of their works studied the effect of porosity and surface chemistry on the adsorption of two VOCs (benzene and toluene) at low concentrations. The results show that in terms of porosity, the volume of narrow micropores (size < 0.7 nm) seems to govern the adsorption of VOCs at low concentration, especially for benzene. The adsorption capacities achieved are higher than previously shown in the literature for these conditions, especially for toluene. Adsorption capacities up to 34 g benzene/100 g CA (340 mg/g CA) or 64 g toluene/100 g CA (640 mg/g CA) have been achieved.

On the other hand, zeolites are widely used as adsorbents due to the properties they present, among which hydrophobicity, adjustable porosity and large surface area (between 250 and 800 m^2^/g) stand out, besides being non-flammable. Zeolites possess superior hydrothermal and chemical stability compared to carbon-based materials [17]. Lee et al. [18] studied the adsorption and desorption behavior of toluene and acetone on a bed of de-luminated Y-zeolite at 20 °C, where the results obtained show that Y-zeolite can be reused without presenting a significant decrease in the adsorption of VOCs from several regeneration cycles.

However, an important new class of porous materials called metal–organic frameworks (MOFs) has been studied as a promising alternative for VOC adsorption. MOFs are crystalline porous materials consisting of an organic ligand and a metal ion or a cluster forming a periodic lattice structure. These materials are characterized by their high specific surface area, chemical functionality and highly ordered crystalline structure [19,20,21,22,23,24]. Open metal sites on the pore surfaces of MOFs can also be available to enhance the adsorption of numerous VOCs. Unlike conventional adsorbents, MOFs maintain their crystalline structure and order after regeneration [25]. Some MOFs have been tested as VOC adsorbents with satisfactory results, including studies on the separation of hydrocarbons on MOFs [26], the controlled reducibility of a MOF with coordinately unsaturated sites for preferential adsorption of gases [27] and the preparation of a defective UiO-66 MOF using MOF-5 as a structural modifier with increased toluene gas sorption capacity [4], among others [25,28,29,30].

In this work, a material called CIM-80 (CIM = Canary Institute Materials) [25], formed by the Al^3+^ cation and the mesaconate ligand, in which water is used as solvent and urea as mild deprotonation reagent and modulator, has been synthesized by means of a green chemistry procedure. This material has also been named by other scientists as MOF Al-MIL-68-Mes [31] in which NaOH has been used instead of urea. Thus, we have studied the synthesis conditions necessary to achieve high synthesis yields (85%) and materials with high crystallinity and accessible porosity. To achieve these results, we have varied the amount of urea in the synthesized materials, also allowing the control of the morphology at the mesoscale level, obtaining more rounded crystals. This material presents a structure similar to that reported by Reinsch et al. [31] and Rocío Bautista et al. [32] in their work. The structure is a lattice with two types of channels, hexagonal pores (ᴓ = 0.6 nm) and triangular pores (ᴓ = 0.2 nm), with the methyl groups of mesaconic acid oriented toward the hexagonal pores. It has a specific surface area B.E.T. of approximately 800 m^2^/g and has potential applications in the extraction and separation of organic compounds. This MOF has been chosen mainly because the porosity range is adequate, since they are ultramicropores, the surface chemistry is also suitable, and of course it is very stable, and its synthesis meets the requirements of the so-called green chemistry. Therefore, once the synthesis of the material was controlled, its behavior in the adsorption/desorption of VOCs, including toluene, m-xylene and cyclohexane, was studied. This study was carried out by means of vapor adsorption isotherms.

## 2. Materials and Methods

All reagents used have been supplied by Sigma Aldrich (St. Louis, MO, USA), in the highest purity available, and none have undergone a subsequent purification process.

The synthesis of the aluminum mesaconate was developed following the procedure described by Adrian Gutierrez-Serpa [33]. For this purpose, a mixture of mesaconic acid (99%) (4 mmol; 520 mg) and aluminum nitrate nonahydrate (98%) (4 mmol; 1500 mg) in 60 mL of deionized water (18.2 MΩ.cm Millipore^®^ Milli-Q^®^ water, Merck, Darmstadt, Germany) with urea (99%) (2 mmol to 10 mmol) was made with constant stirring for 20 min. Subsequently, the solution is transferred to a 100 mL Teflon autoclave lined with a stainless-steel jacket and placed in an oven in which treatment is carried out at 150 °C for 24 h. After 24 h, the autoclave is removed from the oven and cooled to room temperature. Then the white product obtained is separated by filtration and washed repeatedly with distilled water. Finally, it is dried at 50 °C.

### Characterization

Crystallographic phases were identified by powder X-ray diffraction (PXRD) recorded on a Panalytical Empyrean multifunctional equipment for X-ray diffraction analysis, which in its basic configuration has a goniometer with X-ray tube with Cu Kα cathode and Ni filter. It was operated in an angular scanning range of 3° to 40° at an angular velocity of 1°/min and room temperature.

The textural parameters were characterized by means of N_2_ adsorption–desorption isotherms. Samples were subjected to a degassing treatment at 250 °C for 8 h prior to the adsorption measurements. The nitrogen adsorption–desorption isotherms were measured at −196 °C in a Quadrawin (Quantachrome) device. Surface area was determined from the N_2_ adsorption branch. In all cases, the number of points used to apply the BET equation was higher than 5, and the value of c was always positive. Micropore volume (V_micro_) was estimated by the Dubinin–Raduskevich method, and for the pore size distribution, the non-local density functional theory (NLDFT) has been used.

The morphology of samples was studied by Field Emission Scanning Electron Microscopy with X-ray microanalysis (SEM–EDS) (ZEISS-Merlin VP Compact, BRUKER-Quantax 400) in both Backscattered Electron (BSE) and Secondary Electron (SE) modes. Some samples have also been analyzed by Transmission Electron Microscopy (TEM) (JEOL-JEM-1400 Plus).

The characterization of the samples by means of TGA has been carried out in thermogravimetric analysis equipment SDT2960, Simultaneous DSC-TGA, TA Instruments. The experiment was carried out in an Ar atmosphere (100 mL/min) with a heating ramp of 10 °C/min up to a temperature of 1000 °C in an alumina crucible.

The different vibrational modes were studied by Fourier Transform Infrared Spectroscopy using a JASCO IRT-5200 FTIR microscope with a 6× Cassegrain objective and MCT detector (7000–600 cm^−^^1^).

The VOC adsorption/desorption study of VOCs on the MOF was performed by vapour adsorption isotherm on the Vstar of Quantachrome Instruments. This instrument is a volumetric sorption system designed for the analysis of water and organic compounds at sub-atmospheric pressures. It was developed and designed based on the research by the Advanced Materials Laboratory group (LMA) at the University of Alicante. Samples were degassed for 4 h at 250 °C prior to each isotherm measurement. Helium was used to estimate the dead volume, assuming that it is not adsorbed by any of the samples. Adsorption was performed at 25 °C for toluene, cyclohexane and m-xylene.

## 3. Results and Discussion 

### 3.1. Reaction Yield

**Figure 1:**
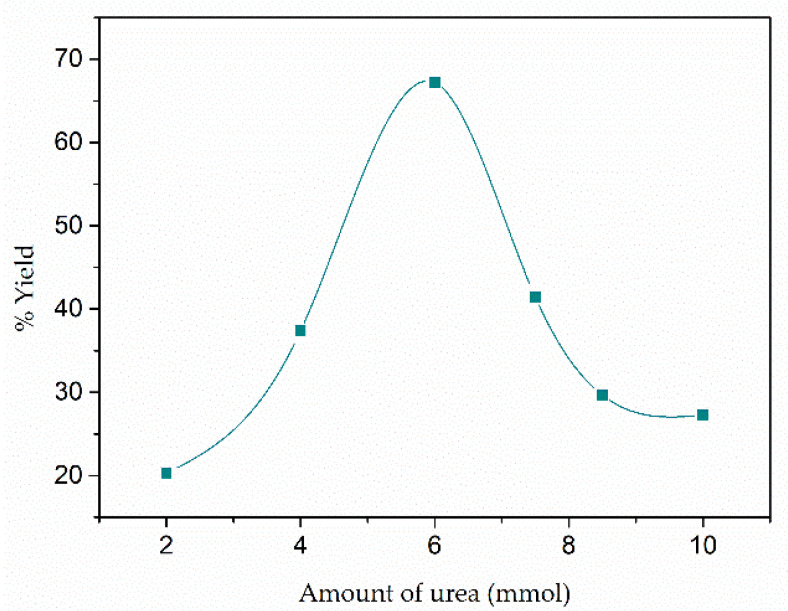
Reaction yield of synthetized materials versus amount of urea.

To optimize MOF synthesis and achieve a high reaction yield, eight materials, in which the amount of urea was varied from 2 mmol to 10 mmol and the amount of aluminum nitrate, mesaconic acid and deionized water were kept constant, were synthesized. The nomenclature of the samples, the amount of urea used, and the reaction yield achieved is shown in Table 1 and Figure 1. With the increase of urea in the synthesis, it is evident how the yield of the reaction increases, but this only occurs up to the “*optimum amount*” of urea, which is 6 mmol. However, as will be seen in the results of the characterization of the materials, from 8.5 mmol of urea, a new phase is formed, different from that of the MOF CIM-80, identified as boehmite. The previously mentioned behavior is due to the fact that urea helps the deprotonation of mesaconic acid only up to the mentioned optimal amount of urea (6 mmol); when adding a higher amount of urea, the pH of the dissolution becomes so basic that it favors the formation of the new phase (boehmite).

Additionally, we synthesized the sample AlO(OH) (boehmite) from aluminum nitrate, urea and water as solvent without adding mesaconic acid. This was performed to compare this sample with the samples containing 10 mmol of urea (CIM-80_10 and CIM-80_10_CTAB) and to verify that the phase formed corresponded to boehmite.

### 3.2. X-ray Diffraction

**Figure 2:**
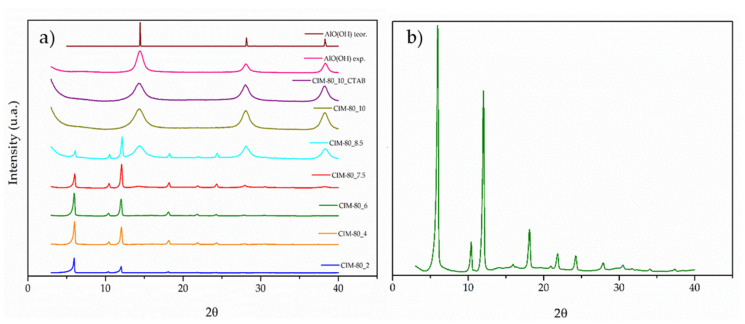
(**a**) X-ray diffraction pattern of the synthesized material; and (**b**) diffraction pattern of the CIM-80_6 sample.

The crystallinity of the synthesized materials was examined by X-ray diffraction in the 2θ range of 3–40° (XRD), as shown in Figure 2. The peaks identified for samples CIM-80_2, CIM-80_4, CIM-80_6 and CIM-80_7.5 are characteristic of MOF CIM-80, as reported in previous studies [32,33]. The samples CIM-80_10 and CIM-80_10_CTAB present broader diffraction peaks, which indicate some decrease in particle size and less crystallinity in the material obtained; these peaks as reported in the literature [34] are characteristic of boehmite (AlO(OH)). Finally, for the sample CIM-80_8.5, it is observed that the material presents the characteristic peaks of the two phases: MOF and boehmite.

### 3.3. Textural Properties—N_2_ Adsorption

**Figure 3:**
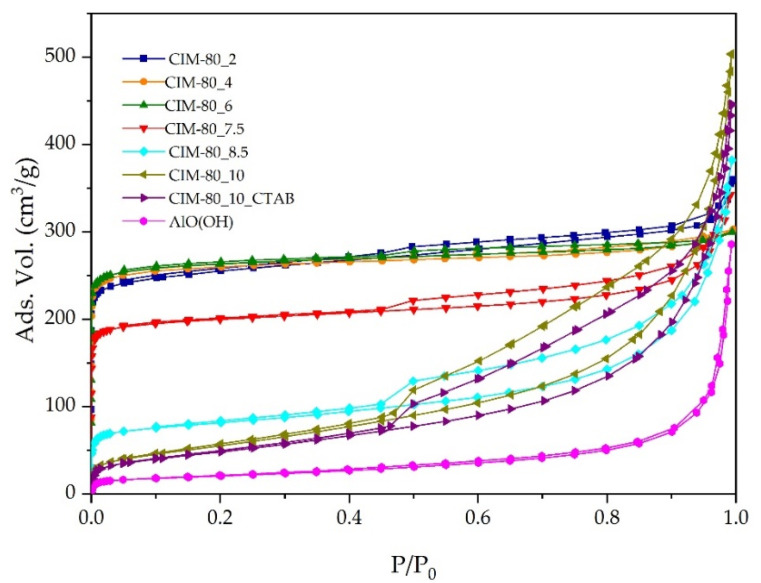
N_2_ adsorption isotherms for the synthesized materials.

The specific surface area of the synthesized materials is calculated using the Brunauer–Emmett–Teller (BET) [35] model from the nitrogen adsorption isotherm (Figure 1 and Figure 3). Samples CIM-80_2, CIM-80_4, CIM-80_6 and CIM-80_7.5 showed type I isotherms [36], characteristic of microporous solids. Samples CIM-80_8.5, CIM-80_10 and CIM-80_10_CTAB present type IV isotherms [36], characteristic of mesoporous adsorbents. The resulting AlO(OH) isotherm provides information of a non-porous solid, which adsorbs only the external surface of the material.

Table 2 compiles the textural properties of all the samples. Here, it can be observed that as the amount of urea in the syntheses (samples CIM-80_2, CIM-80_4, CIM-80_6) increases, the specific surface area is larger as well as the volume of micropores. From sample CIM-80_7.5 (7.5 to 10 mmol urea), the specific surface area decreases as well as the micropore volume, increasing the mesopore volume. 

**Figure 4:**
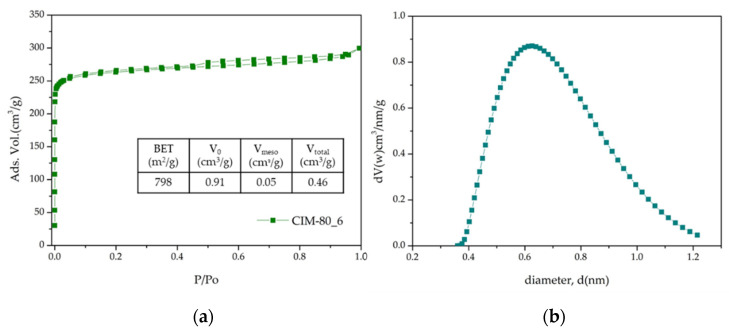
(**a**) N_2_ adsoprtion isotherm; (**b**) pore size distribution of sample CIM-80_6.

Figure 4a shows the nitrogen adsorption isotherm for sample CIM-80_6, which presents the highest specific surface area, approximately 800 m^2^/g; this value was close but a little lower than the value reported by Rocío-Bautista et al. [32]. The isotherm is type I, characteristic of microporous solids. At low relative pressures, a rapid increase of the adsorbed quantity is observed (250 cm^3^/g), and the micropore volume of the sample was 0.41 cm^3^/g. The pore sizes and pore volumes of CIM-80_6, i.e., the pore size distribution was estimated by NLDFT and are shown in Figure 4b. The calculated mean pore diameter was 0.63 nm, and the cumulative pore volume was 0.87 cm^3^/g.

### 3.4. Scanning Electron Microscopy (SEM) and Transmision Electronic Microscopy (TEM) 

**Figure 5:**
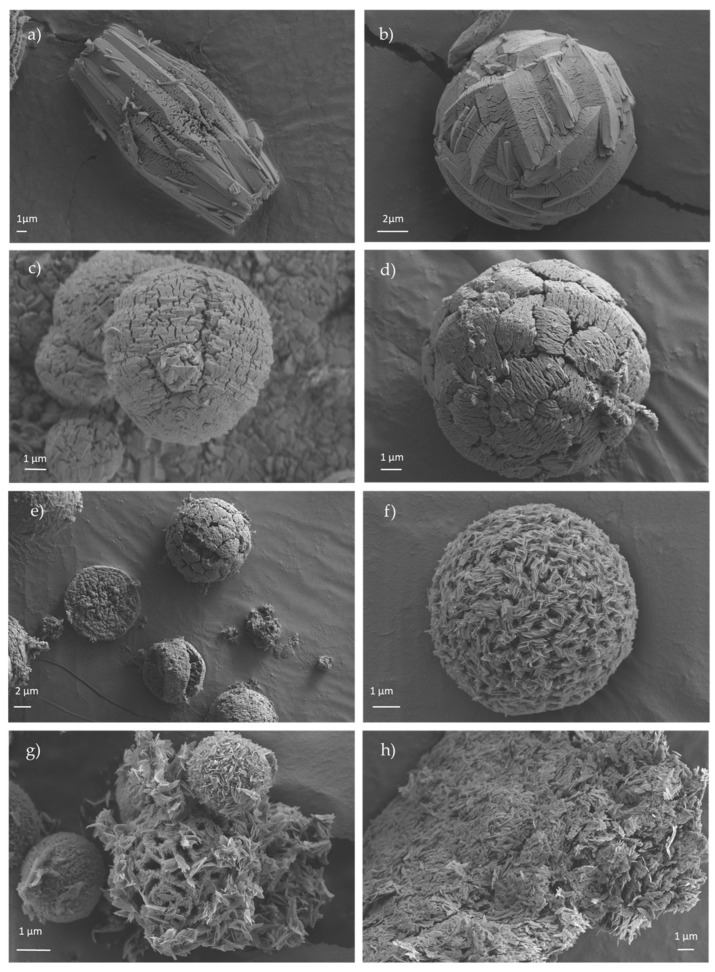
FESEM micrographs of the synthesized materials: (**a**) CIM-80_2; (**b**) CIM-80_4; (**c**) CIM-80_6; (**d**) CIM-80_7.5; (**e**) CIM-80_8.5; (**f**) CIM-80_10; (**g**) CIM-80_10_CTAB; (**h**) AlO(OH).

**Figure 6:**
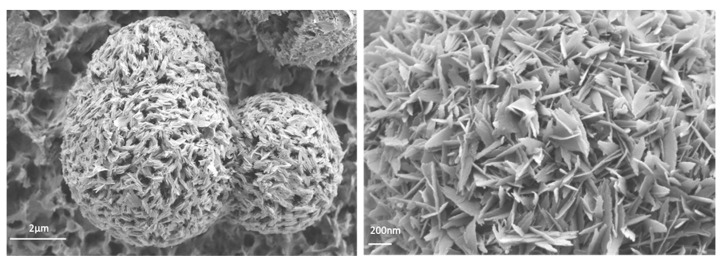
CIM-80_8.5 FESEM micrographs.

**Figure 7:**
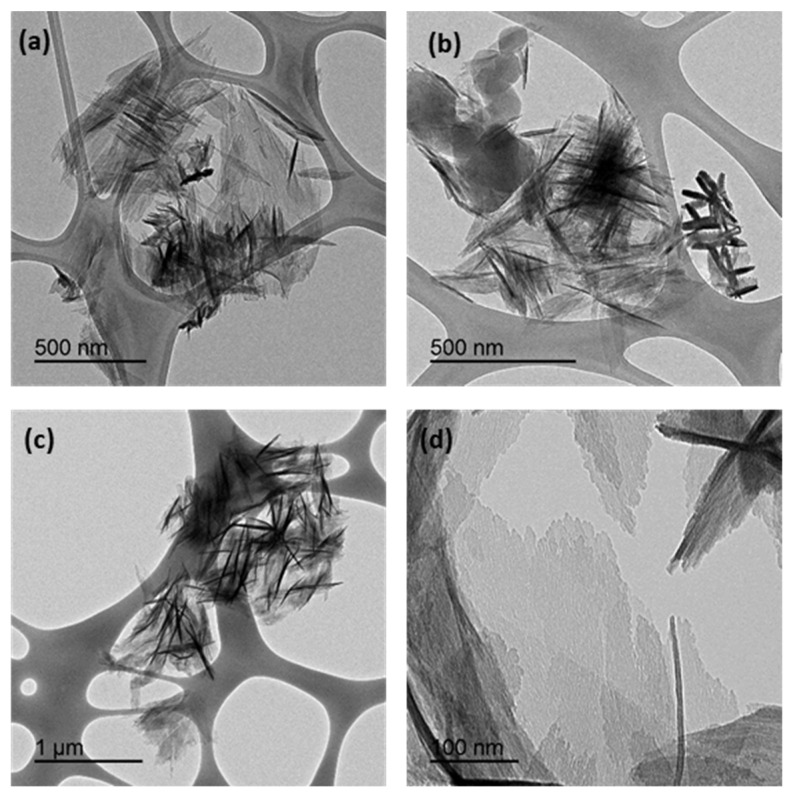
TEM micrographs of the synthesized materials: (**a**) CIM-80_8.5; (**b**) CIM-80_10; (**c**) CIM-80_10_CTAB; (**d**) AlO(OH).

Figure 5 shows the FESEM images of the crystals obtained for the eight samples synthesized under different amounts of urea. Apparently, the shape of the crystals can be easily adjusted by varying the amount of urea, which acts as a mild deprotonation agent. For sample CIM-80_2 (Figure 5a), elongated crystals with average lengths of about 22 μm were obtained. By increasing the amount of urea, it is observed how the crystals adopt a rounded shape (Figure 5b–e) with diameters of about 13 μm, 8.6 μm, 12 μm and 8.5 μm, respectively. In sample CIM-80_8.5, the clustering of the elongated particles in a circular shape leaves empty spaces between the particles, resulting in the formation of a larger volume of mesopores (0.47 cm^3^/g, V_total_: 0.59 cm^3^/g) than that of the samples prepared with less urea (Figure 6). In the case of sample CIM-80_10, elongated and flat crystals (5 μm × 0.11 μm, in length and width) were obtained that agglomerate into apical forms. CIM-80_10_CTAB was prepared adding CTBA in the synthesis with the aim of studying the effect it could exert as a shape director in the material as other scientists have reported for other types of materials [30,31,32]. However, the result was not satisfactory since, as shown in Figure 5g, the agglomeration of the crystals is less homogeneous than the sample without CTAB containing the same amount of urea (Figure 5f). Finally, Figure 5h presents the morphology of the AlO(OH) sample formed by flat and elongated crystals, with approximate lengths of 0.72 μm. Particles from samples CIM-80_8.5, CIM-80_10, CIM-80_10_CTAB and AlO(OH) were also characterized by TEM (Figure 7). For the four samples, particles in the form of lamellae with well-defined edges can be observed agglomerating with each other. The sizes of the lamellae range from 0.5 to 3 μm.

Taking into account that boehmite is a precursor of γ-alumina [37], which is used as catalyst supports, an additional study was performed in which CIM-80_10 and AlO(OH) samples were calcined at 550 °C and characterized by XRD, N_2_ adsorption and FESEM techniques (see figures and tables in the section “*characterization of calcined samples*”) to check if it was possible to obtain γ-alumina from the boehmite obtained in the samples containing 10 mmol of urea in their synthesis.

### 3.5. Thermogravimetric Analysis (TGA) and Fourier Transform Infrared Spectroscopy (FTIR) 

**Figure 8:**
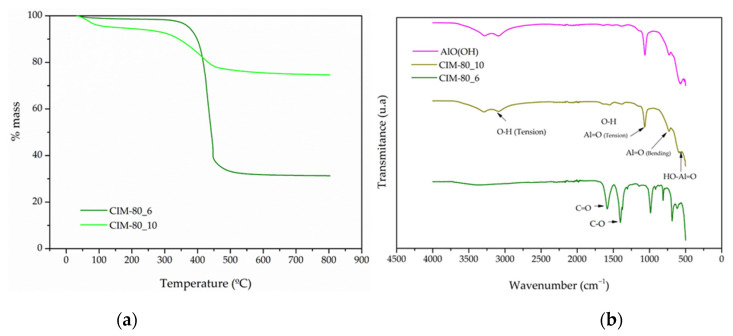
(**a**) Thermogravimetric analysis (TGA) curves in a nitrogen atmosphere of CIM-80_6 and CIM-80_10 samples; (**b**) FTIR spectra for the CIM-80_6, CIM-80_10 and AlO(OH) samples.

Figure 8a shows the weight loss of CIM-80_6 and CIM-80_10 samples measured by thermogravimetric analysis (TGA) in an Ar atmosphere from 25 °C to 800 °C. The CIM-80_6 material showed a single stage of weight loss of approximately 70% at a heating temperature below 400 °C, which is attributed to decomposition of the CIM-80. The CIM-80_10 showed two weight-loss steps. For the first step, approximately 10% of weight was lost by heating to 25 °C to 100 °C, which is attributed to adsorbed water molecules. The second step was about 25% or the weight at a heating temperature below 300 °C, which is attributed to decomposition of the material.

2AlO(OH) → γ-Al_2_O_3_ + H_2_O↑

The FTIR spectra shows the bands for the CIM-80_6, CIM-80_10 and AlO(OH) (Figure 8b). In sample CIM-80_6, the band corresponding to the C=O group stress vibration appears as a strong band at 1585 cm^−^^1^. At 1402 cm^−^^1^, a strong intensity band with an adjacent shoulder at 1374 cm^−^^1^ corresponding to the C-O stress vibration is observed. The band corresponding to the O-H group stress vibration observed for mesaconic acid between 2587 and 2827 cm^−^^1^ is a broad band due to hydrogen bridge formation formed by two low intensity peaks. This band disappears in the spectrum of all samples as mesaconic acid reacts to form mesaconate.

For the CIM-80_10 and AlO(OH) samples, the same bands are present throughout the spectrum. The band corresponding to the bending vibration of the O-H group appears at 1154 and 1146 cm^−^^1^ for CIM-80_10 and AlO(OH), respectively, and is characteristic of boehmite [38]. This band shows a shift toward lower wave values and may be due to more involvement of the amorphous phase. Such a band is usually presented as a shoulder masked by a stronger band corresponding to the Al=O stress mode at 1068 cm^−^^1^ [38]. For the two samples analyzed, a slight shift toward lower values located at 1065 and 1063 cm^−^^1^ is also observed. The bands corresponding to the Al=O bending vibration appear at 729 and 731 cm^−^^1^. The band corresponding to bending in the plane of the HO-Al=O angle, bands appear between 598–578 cm^−^^1^ and between 609–580 cm^−^^1^, whose positions coincide with the amorphous boehmite vibrational modes [39]. In samples CIM-80_10 and AlO(OH), the stretching band of the OH group, which is attributed to adsorbed water molecules, is observed between 3000 and 3200 cm^−^^1^. This band does not appear in the spectrum of sample CIM-80_6, reflecting the hydrophobic character of the material.

### 3.6. Characterization of Calcined Samples

**Figure 9:**
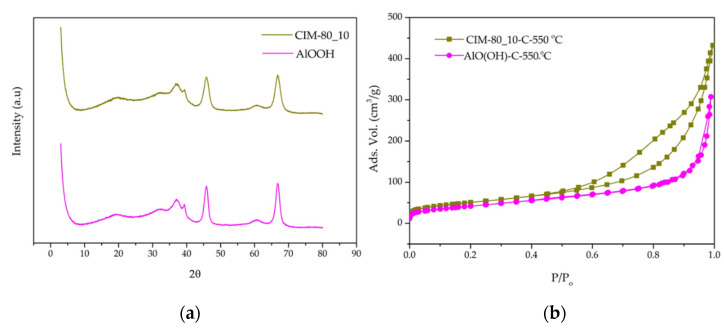
CIM-80_10-C-550 °C and AlO(OH)-C-550 °C samples: (**a**) X-ray diffraction patterns; (**b**) N_2_ adsorption isotherms.

For the samples calcined at 550 °C, the results obtained coincide with the properties of a transition alumina identified as γ-Al_2_O_3_ [40], which presents a spinel-type structure. Figure 9a shows the diffraction peaks of the two samples, identified at θ = 20.15°, 36.55°, 39.35°, 46° and 66.85°, which correspond to the mentioned alumina phase (JCPDS 29-0063). The adsorption isotherms of N_2_ at −196.15 °C are shown in Figure 9b. The isotherm obtained for the sample CIM-80_10-C-550 °C is type IV with an H3 hysteresis cycle; this type of isotherm is characteristic of a mesoporous solid (sample V_meso_: 0.59 cm^3^/g). This isotherm coincides with the results obtained by other scientists for the γ-Al_2_O_3_. For the sample AlO(OH)-C-550 °C, the isotherm obtained is characteristic of non-porous solids, which adsorb only on the external surface of the material. Table 3 shows the textural properties obtained from the N_2_ adsorption isotherms of the two samples analyzed. The two materials present specific surfaces of similar size (181 y 150 m^2^/g), and the pore volume for the two samples corresponds mostly to mesopores.

**Figure 10:**
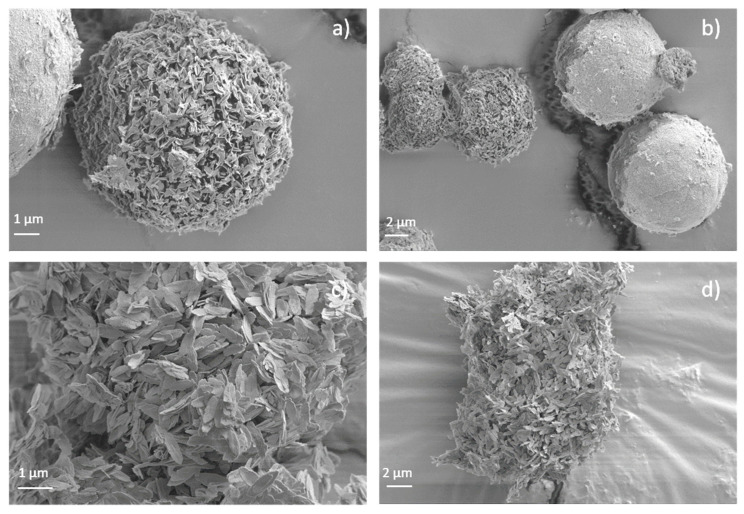
FESEM micrographs of the samples calcined at 550 °C: images (**a**,**b**) correspond to CIM-80_10-C-550 °C and images (**c**,**d**) correspond to AlO(OH)-C-550 °C.

Figure 10 shows the FESEM image of the powder of the calcined samples. The powder of the two samples CIM-80_10-C-550 °C and AlO(OH)-C-550 °C consists of nanofibers of about 0.5 m and 2 mm in length, respectively. These nanofibers tend to form ball-shaped agglomerates for sample CIM-80_10 (which contains mesaconic acid) and amorphous agglomerates for sample AlO(OH) due to their high surface energy, thus generating high porosity. 

### 3.7. Water and VOC Adsorption on MOF CIM-80_6

**Figure 11:**
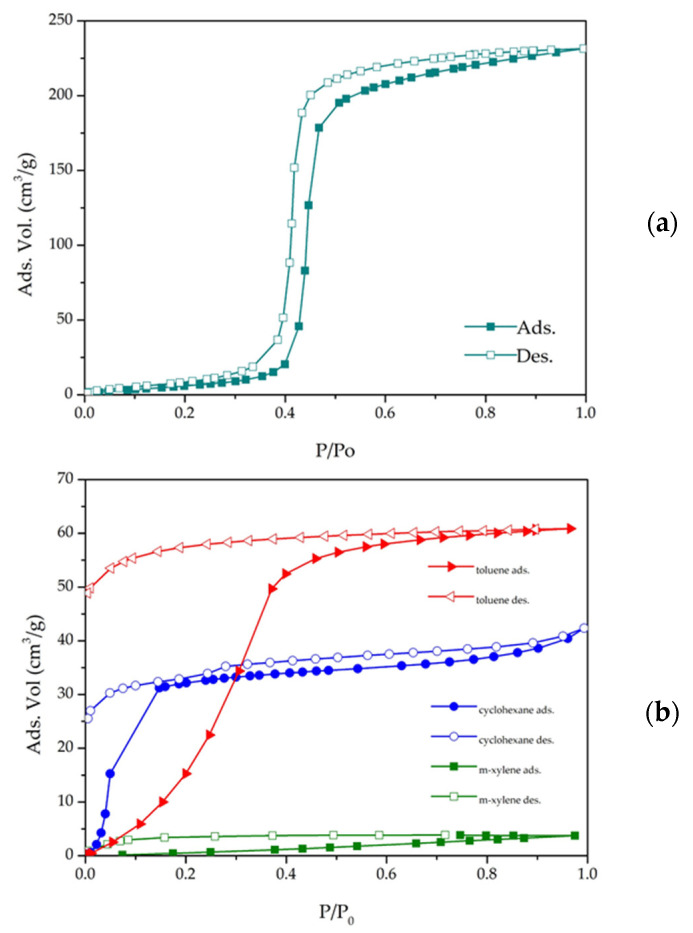
(**a**) Isotherm of water at 25 °C; (**b**) isotherms of toluene, cyclohexane and m-xylene at 25 °C.

Figure 11a shows the water adsorption behavior of the CIM-80_6 material. The water vapor isotherm shows a sinusoidal shape. This type of adsorption isotherms, which is called Type V in the IUPAC classification, is observed when the fluid–solid interaction is weak compared to the fluid–fluid interaction, as in the case of water adsorption in some activated carbon [41] and some MOFs that exhibit stability in water such as Al-MIL-68-Mes [31], Cr-MIL-101, Zr-UiO-66-NH2, Ti-MIL-125 and In-MIL-68 [41]. The opposite case occurs for materials that present affinity for water, such as hydrophilic zeolites and some MOFs as Ga-MIL-53 [42] and Al-MIL-53-NH_2_ [41]; their isotherms are of type I and are characterized by a strong adsorption at low pressure followed by a large saturation plateau.

Figure 11a shows an increase in adsorption around P/P_0_ = 0.45 from 50 cm^3^/g (45.76 mg/g) to 270 cm^3^/g (231.45 mg/g) that reaches a maximum adsorption capacity of 290 cm^3^/g at a P/Po = 1. The water isotherm results support the FITR results, which show the hydrophobic character of the material. Figure 11b shows the adsorption isotherms of the VOCs (toluene, cyclohexane and m-xylene) at 25 °C. Toluene is the compound for which MOF showed the highest adsorption capacity, with 60.74 cm^3^/g (250.6 mg/g) being the maximum amount adsorbed at a P/P_0_ = 0.8. The maximum amount of cyclohexane adsorbed was 42.34 cm^3^/g (159 mg/g) at a P/P_0_ = 1, followed by m-xylene, for which the maximum adsorption was 3.86 cm^3^/g (18.29 mg/g). It is important to point out different aspects of the curves, especially if we compare cyclohexane and toluene, since xylene absorbs very little; both only desorb 10%, which indicates the great affinity with the surface. In addition, the adsorption of cyclohexane starts much earlier despite being larger in size due to the higher affinity for the methyl of the MOF.

Table 4 lists the following properties of the VOCs studied: density, kinetic diameter (D), molecular cross-sectional area (σ), quantity of VOC adsorbed on the MOF (Q_0_) and adsorption capacity in volume (V).

**Figure 12:**
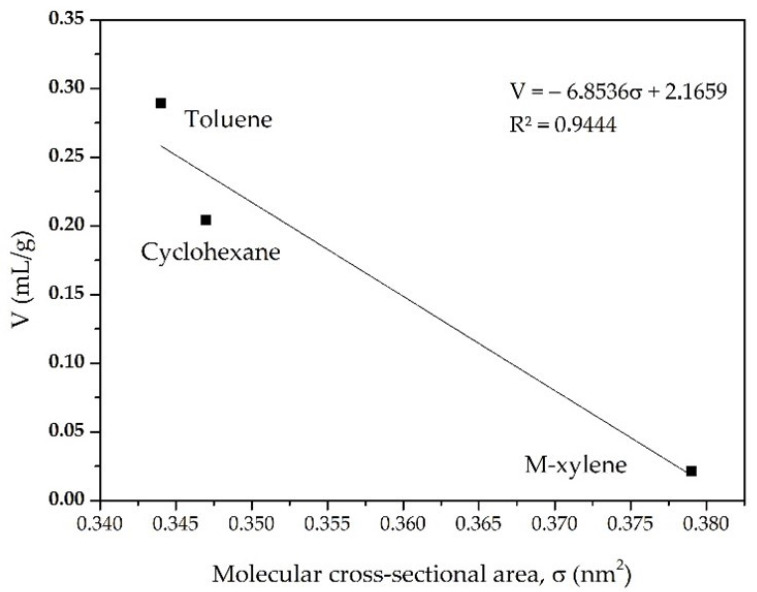
Volume adsorption capacity (V) of VOCs on MOF CIM-80 versus the molecular cross-sectional areas (σ) of VOCs.

Figure 12 shows the amount of VOC adsorbed on CIM-80_6 versus the size of the molecular cross-sectional area. We can see the directly proportional relationship between the size of the molecule and the amount adsorbed on the MOF, with the toluene molecule being the smallest (0.344 nm^2^) and the one that is mostly adsorbed by the MOF (0.289 mL/g). On the other hand, the cyclohexane molecule is slightly larger than the toluene molecule (0.347 nm^2^), and the amount adsorbed is slightly less (0.204 mL/g). Finally, the m-xylene molecule is the largest (0.379 nm^2^) and is the one that is less able to be adsorbed (0.021 mL/g) by the MOF. Taking into account that the MOF obtained has pores of 0.2 nm and 0.6 nm in diameter, the adsorption behavior of the VOCs in the MOF is as expected, and the molecules being adsorbed pass through the hexagonal channels (0.6 nm in diameter). Therefore, the m-xylene having a molecule size larger than the largest pores of the MOF makes it difficult to be adsorbed.

Table 5 shows the adsorption capacity of porous materials, including activated carbon, zeolite and MOF, as well as the MOF studied in this work (CIM-80). The amount of toluene adsorbed on CIM-80 is significantly lower (250.6 mg/g) than that reported for some activated carbons (640 mg/g) [16] and some MOFs (MIL-177 (585 mg/g)) [28]. In the case of AC and MOF-177, these materials have a higher specific surface area (2476 and 2970 m^2^/g, respectively) than MOF CIM-80 (798 m^2^/g) in addition to having a larger pore volume (0.97, 1.11 and 0.63 cm^3^/g, respectively). On the other hand, MIL-125-NH_2_ has an adsorption capacity of 293 mg/g [28] and a B.E.T. surface area of 1280 m^2^/g. With respect to CIM-80, its adsorption capacity is a little higher; it has been reported that the amino group in the MOF imparts an additional adsorption capacity on the MOF [38]. This behavior is also reflected in the amount of toluene adsorbed on UiO-66 and UiO-66- NH_2_. In this order, the adsorption capacity of CIM-80 is in the order of MIL-125-NH2 and is higher than the adsorption capacity of UiO-66, ZIF-67 and MOF-5 (64.2, 50.8 and 32.9 mg/g, respectively). It is also above the adsorption capacity of Zeolite 13× (16.03 mg/g) and some activated carbons. Taking into account that the compared materials that have been synthesized by procedures that are not so environmentally friendly (use of organic solvents, very acidic or very basic preparations), the material analyzed in this study is a promising alternative in the adsorption of volatile organic compounds, thanks to its properties and easy and environmentally friendly synthesis procedure. CIM-80 has very narrow, well-defined pores and has a very hydrophobic character, which makes it have a great potential for hydrocarbon separation. In our research group, we are performing preliminary studies on this topic, and they will probably be published soon.

## 4. Conclusions

MOF CIM-80 was successfully synthesized under sustainable conditions, optimizing the amount of reagents needed (6 mmol of urea) to obtain higher reaction yields, crystallinity and mesoscale control. The material obtained has a high specific surface area of 800 m^2^/g, in addition to presenting thermal (350 °C) and water vapor adsorption stability, which in turn confirms the hydrophobic character of the MOF, a condition that makes it potentially interesting in adsorption applications of volatile organic compounds, among which toluene and cyclohexane stand out (250.6 mg/g and 159 mg/g). The m-xylene is the VOC least adsorbed (18.29 mg/g) by MOF. This work provides a research basis to study the adsorption characteristics of VOCs on MOF CIM-80.

The samples whose formed phase was identified as AlO(OH) of pseudoamorphous character low porosity (between 0.03 and 0.08 cm^3^/g) and low specific surface area (between 72 and 205 m^2^/g) were subjected to a calcination process at 550 °C, which resulted in a material whose crystallographic, textural and morphological properties correspond to those of γ-Al_2_O_3_. Taking into account that γ-Al_2_O_3_ is a material that is widely used as a catalyst support, this type of synthesis can be a viable alternative for its production. In particular, the use of mesaconic acid is of great interest as it allows us to control the morphology at the mesoscale level.

## Figures and Tables

**Table 1:** Reaction yield obtained in each synthesized material. The nomenclature of the samples is composed by the MOF name_amount of urea used in the synthesis. CTAB = to cetyltrimethylammonium bromide.

Sample	Amount of Urea	% Yield
CIM-80_2	2 mmol	20
CIM-80_4	4 mmol	37
CIM-80_6	6 mmol	67
CIM-80_7.5	7.5 mmol	41
CIM-80_8.5	8.5 mmol	30
CIM-80_10	10 mmol	27
CIM-80_10_CTAB	10 mmol + CTAB	27
AlO(OH)	10 mmol	98

**Table 2:** Textural properties obtained by the N_2_ isotherms.

Sample	Specific Surface BET (m^2^/g)	V_0_ (cm^3^/g)	V_meso_ (cm^3^/g)	V_total_ (cm^3^/g)
CIM-80_2	787	0.39	0.17	0.56
CIM-80_4	787	0.40	0.06	0.46
CIM-80_6	798	0.41	0.05	0.46
CIM-80_7.5	610	0.31	0.22	0.53
CIM-80_8.5	265	0.12	0.47	0.59
CIM-80_10	205	0.08	0.70	0.78
CIM-80_10_CTAB	177	0.07	0.62	0.69
AlO(OH)	72	0.03	0.41	0.44

**Table 3:** Textural properties obtained from N_2_ adsorption isotherms of calcined samples.

Sample	BET (m^2^/g)	V_o_ (cm^3^/g)	V_meso_	V_total_
CIM-80_10-C-550 °C	181	0.08	0.59	0.67
AlOOH-C-550 °C	150	0.06	0.42	0.47

Nomenclature of the samples: name of sample_amount of urea-C(Calcined)-Temperature of calcined.

**Table 4:** Properties of selected VOCs.

VOCs	Density, ρ (g/mL, 25 °C)	M (g/mol)	D (nm)	σ (nm^2^)	Q_0_ (mg/g)	V (mL/g)
Toluene	0.866	92.15	0.585	0.344	250.26	0.289
Cyclohexane	0.779	84.16	0.600	0.347	159	0.204
m-xylene	0.860	106.16	0.680	0.379	18.29	0.021

D: kinetic diameter; from reference [43]: σ: molecular cross-sectional area; calculated using the equation: σ=1.091∗(MρN)23, N: Avogadro number; from Ref. [44]. V: volume adsorption capacity, calculated from Q_0_ and density; density data from reference [28].

**Table 5:** Toluene adsorption capacities: comparison CIM-80 with other materials.

Material	Precursor	B.E.T. (m^2^/g)	V_total_ (cm^3^/g)	V_0_ (cm^3^/g)	Pore Size (nm)	Green Synthesis	Q_0_(mg/g) Toluene	Ref
AC	Anthracite	2746	0.97	0.8	-	↓ ^B^	640	[16]
AC	Organic biomass	990	-	0.09	2.7	↓ ^B^	109	[45]
AC	Organic biomass	805	-	0.47	2.35	↓ ^B^	59.2	[46]
Zeolite 13×	Si/Al	440	0.12	-	9.9	↓ ^B^	16.02	[45]
MIL-100	Fe	1398	1.08	0.46	1.36	↑ ^WS^	663	[47]
MIL-101	Cr	3980	1.85	-	2.6	↑ ^WS^	1067	[44]
MOF-177	Zn	2970	1.11	-	0.94	↓ ^OS^	585	[28]
MIL-125-NH_2_	Ti	1280	-	0.56	0.4	↓ ^OS^	293	[48]
UiO-66-NH_2_	Zr	1250	0.62	-	1.98	↓ ^OS^	147	[49]
UiO-66	Zr	1414	0.68	-	1.93	↓ ^OS^	64.2	[49]
ZIF-67	Co	1401	1.22	-	3.49	↑ ^WS^	50.8	[49]
MOF-5	Zn	424	0.22	-	2.07	↓ ^OS^	32.9	[49]
CIM-80	Al	798	0.46	0.41	0.63	↑ ^WS^	250.6	This work

Green synthesis: refers to how environmentally friendly the synthesis of the material is, depending on: ^B^: highly basic medium, ^OS^: organic solvents, ^WS^: water as solvent. The adsorption studies of the materials presented in this table were performed by TG. The adsorption behavior of our material was studied by vapor adsorption isotherms.

## Data Availability

Not applicable.
